# Estimated Incidence of Sugammadex-Induced Anaphylaxis Using the Korea Adverse Event Reporting System Database

**DOI:** 10.3390/jcm10153202

**Published:** 2021-07-21

**Authors:** Jae-Woo Ju, Nayoung Kim, Seong Mi Yang, Won Ho Kim, Ho-Jin Lee

**Affiliations:** 1 Department of Anesthesiology and Pain Medicine, Seoul National University Hospital, Seoul 03080, Korea; jujw701@naver.com (J.-W.J.); nyk0421@hanmail.net (N.K.); seongmi.yang@gmail.com (S.M.Y.); wonhokim@snu.ac.kr (W.H.K.); 2 Department of Anesthesiology and Pain Medicine, Seoul National University College of Medicine, Seoul 03087, Korea

**Keywords:** adverse drug reaction reporting systems, anaphylaxis, drug-related side effects and adverse reactions, epidemiology, hypersensitivity, sugammadex

## Abstract

We aimed to investigate the incidence of sugammadex-induced anaphylaxis in a large Korean population. We retrospectively investigated the incidence of sugammadex-induced anaphylaxis between 2013 and 2019 from the database of the Korea Institute of Drug Safety-Risk Management-Korea Adverse Event Reporting System (KIDS-KAERS). We estimated the incidence of sugammadex-induced anaphylaxis from the KIDS-KAERS database, assuming that the reporting efficiency was 10%, 50%, and 100%, respectively. We also investigated its annual sales volume in Korea and assumed that the exposure to sugammadex was 95% of the estimated sales volume. During the study period, 1,401,630 sugammadex vials were sold, and 19 cases of sugammadex-induced anaphylaxis were identified in the KIDS-KAERS database. The estimated incidence of sugammadex-induced anaphylaxis was 0.0143%, 0.00279%, and 0.0014%, assuming a reporting efficiency of 10%, 50%, and 100%, respectively. All patients, except for one with a missing record, fully recovered after anaphylaxis. The incidence of sugammadex-induced anaphylaxis identified in the national pharmacovigilance database was lower than previously reported rates in other countries. Therefore, its use in general anesthesia should not be hindered by concerns about the resulting risk of anaphylaxis in Korea.

## 1. Introduction

Sugammadex is a modified γ-cyclodextrin that reverses the effect of steroidal non-depolarizing neuromuscular blocking agents (NMBAs) by forming a sugammadex-NMBA complex at a ratio of 1:1 [1]. Sugammadex is associated with faster neuromuscular recovery, a lower rate of residual neuromuscular blockade [2], a lower incidence of major pulmonary complications [3], and earlier first bowel movement after surgery [4], compared to anticholinesterase inhibitors, which are conventional NMBA reversal agents. Reversal of neuromuscular blockade with sugammadex is also associated with a lower incidence of 30-day unplanned readmission, shorter hospital stays, and reduced hospital charges [5]. Therefore, sugammadex has gained popularity among anesthesiologists and is rapidly replacing conventional reversal agents [6].

However, concerns over life-threatening hypersensitivity reactions associated with sugammadex have been raised, which has delayed its approval by the United States Food and Drug Administration (FDA) by approximately 8 years [7]. In a randomized controlled study conducted by the manufacturer (Merck Sharp & Dohme (MSD)) to respond to the FDA’s request, one in 375 healthy volunteers experienced sugammadex-induced anaphylaxis after receiving an injection of sugammadex of 16 mg kg^−1^ [8]. Subsequently, another study conducted by the manufacturer showed no difference in the incidence of hypersensitivity or anaphylaxis between sugammadex and neostigmine or placebo [9]. Additionally, a total of 273 anaphylaxis cases were identified in the post-marketing database, with approximately 11.5 million cases of sugammadex exposure [9]. Subsequently, the FDA approved its use in December 2015.

Since then, the incidence of sugammadex-induced anaphylaxis in a large-scale population has been reported for a few countries, but not for South Korea [6,10,11,12]. According to the pharmacopoeia of sugammadex (Bridion^®^, MSD, Seoul, Korea) in South Korea, no anaphylactic reaction has been reported in a post-marketing survey of 718 patients [13], and only a few case reports have been published in South Korea [14,15,16,17,18]. Considering the trend of its rapidly increasing use for anesthesia, it is meaningful to investigate the incidence of sugammadex-induced anaphylaxis in a large-scale population. To this end, we investigated the nationwide number of reported cases of sugammadex-induced anaphylaxis using the database of the Korea Institute of Drug Safety & Risk Management-Korea Adverse Event Reporting System (KIDS-KAERS). We also investigated the incidence of sugammadex-induced anaphylaxis in a tertiary teaching hospital in South Korea.

## 2. Materials and Methods

### 2.1. Study Design

The study protocol consisted of three sections. First, we investigated the cases of patients diagnosed with sugammadex-induced anaphylaxis using the KIDS-KAERS database. Second, we reviewed relevant case reports published in South Korea. Finally, we retrospectively investigated cases of sugammadex-induced anaphylaxis in our institution.

This study was approved by the Institutional Review Board (IRB) of Seoul National University Hospital on 30 March 2021 (No. 2103-166-1207), and the requirement for informed consent was waived since this was a retrospective study.

#### 2.1.1. Analysis of Nationwide Data

In this part, we aimed to investigate the cases of sugammadex-induced anaphylaxis reported in the KIDS-KAERS database; this is a publicly available nationwide database in South Korea that facilitates the reporting and management of ADEs [19]. Anyone who experiences an ADE can report it to the KIDS-KAERS database, and the causal relationships in the ADE reports are evaluated in regional pharmacovigilance centers according to the WHO-UMC international drug monitoring program [19].

We extracted the cases related to the use of sugammadex reported between 2013 and 2019 in the KIDS-KAERS database and categorized them as anaphylactic reaction (World Health Organization—Adverse Reactions Terminology (WHO-ART) 2237-1), anaphylaxis (WHO-ART 2237-2), anaphylactic shock (WHO-ART 713), anaphylactoid reaction (WHO-ART 714), purpura anaphylactoid (WHO-ART 460-3), or anaphylactic vascular purpura (WHO-ART 460-7). Data for 2020 were not available. Since the data were obtained after de-identification, the need for informed consent was waived. The extracted data included age, sex, year of the event, history of drug allergy, underlying diseases, the dosage of sugammadex, other suspected drugs, level of seriousness (namely, clinically important situation, prolonged admission, life-threatening complication, disability, or severe degree of functional decline, and death), recovery status, and the subject of reporting.

Furthermore, we investigated the annual sales volume (unit: vials) of sugammadex in South Korea using the database of the manufacturer (MSD Korea Inc., Seoul, Korea) to estimate its annual usage. Since sugammadex is not covered by national health insurance in South Korea, we had no other means to investigate its annual usage volume. We assumed that each patient used one vial in one operation and the exposure to sugammadex was 95% of the estimated sales volume [20].

#### 2.1.2. Case Reports Published by Korean Researchers

We retrieved case reports related to sugammadex-induced anaphylaxis published by Korean researchers between 2013 and 2020 and available on the Web of Science, Google Scholar, and Scopus databases. The following search terms were used: TS = (sugammadex AND (anaphyl* OR hypersensitivity)) on the Web of Science; anaphylaxis, OR hypersensitivity, OR Korea “sugammadex” on Google Scholar; TITLE-ABS-KEY (sugammadex AND (anaphyl* OR hypersensitivity)) on Scopus. Then, two authors (J.-W.J, H.-J.L.) independently reviewed the titles and abstracts of all searched articles to identify studies related to sugammadex-induced anaphylaxis published by Korean researchers. Cases of patients not diagnosed with sugammadex-induced anaphylaxis were excluded. Details, such as demographics, previous exposure to sugammadex, dosage of sugammadex, onset of anaphylaxis after sugammadex administration, time to achieve hemodynamic stability after anaphylaxis, skin prick test, intradermal skin test, and complications after recovery, were retrieved from the case reports.

To evaluate the reliability of the extracted data from the KIDS-KAERS database, we also investigated whether the patients identified in the searched case reports were also included in the KIDS-KAERS data when matched by age, sex, and year.

#### 2.1.3. Single-Center Retrospective Cohort Study

To investigate the incidence of sugammadex-related anaphylaxis in our institution, we followed the protocol of a recently published study regarding sugammadex-induced anaphylaxis [12]. From the electronic medical records (EMR) database, we selected adult patients (aged ≥18 years) who underwent surgery under general anesthesia with the use of sugammadex for reversal of neuromuscular blockade at the end of the surgery, between January 2013 and December 2020.

Two parallel independent electronic searches were simultaneously performed to meticulously identify cases of sugammadex-induced anaphylaxis in our institution. First, the drug adverse reaction system in the EMR database was reviewed to identify patients who were manually labeled as having a drug allergy to sugammadex. In our institution, physicians and nurses can report previously known or current incidences of adverse drug events (ADEs) to the drug adverse reaction system in the EMR. Second, we searched for anesthetic records where both sugammadex and epinephrine were administered intravenously to the same patient [12].

If patients met either search criteria, a chart review was performed by two independent researchers (J.-W.J. and H.-J.L.) to assess the occurrence of anaphylaxis, according to the World Allergy Organization (WAO) guidelines for the assessment and management of anaphylaxis [21]. The causal relationship was evaluated according to the WHO-Uppsala Monitoring Centre (UMC) international drug monitoring program [22]. Additionally, to evaluate the reliability of our protocol, we also investigated the incidence of rocuronium-induced anaphylaxis in the same patient population using an identical protocol.

### 2.2. Statistical Analysis

Descriptive statistics were conducted using R software version 4.0.0 (R Core Team, 2020. R: Language and Environment for Statistical Computing. R Foundation for Statistical Computing, Vienna, Austria. https://www.R-project.org/, accessed on 14 April 2021). Categorical data are described as percentages, and continuous data as medians and interquartile ranges [IQR]. We estimated the incidence of sugammadex-induced anaphylaxis from the KIDS-KAERS database, assuming that the reporting efficiency was 10%, 50%, and 100%, respectively. For voluntary reporting systems, under-reporting is always a concern, and reporting efficiency is conservatively calculated as 10% [22]. However, the reporting efficiency of severe adverse drug reactions (ADRs) has been reported to be five times higher than that of non-serious events [23]. Anaphylaxis is one of the most severe ADRs; therefore, its reporting efficiency would have been higher than other ADRs.

## 3. Results

### 3.1. Analysis of Nationwide Data

According to the sales data of the manufacturer, the annual sales volume of sugammadex increased by approximately 613% from 57,340 vials in 2014 to 408,860 vials in 2019. During the study period, a total of 1,401,630 vials of sugammadex were sold (500 mg/5 mL, 1340 vials; 200 mg/2 mL, 1,400,290 vials). The estimated population exposure to sugammadex was 1,331,549. In the KIDS-KAERS database, 19 cases of sugammadex-induced anaphylaxis were identified (Table 1). If the reporting efficiencies are assumed to be 10%, 50%, and 100%, the estimated incidence of sugammadex-induced anaphylaxis was 14.3 per 100,000 exposures (0.0143%), 2.9 per 100,000 exposures (0.0029%), and 1.4 per 100,000 exposures (0.0014%), respectively. Of them, 14 (73.7%) patients were male, and the median (IQR) age was 58 (38–62) years. Two patients had a history of drug allergies, and one was diagnosed with asthma. Rocuronium was the most frequent other culprit drug (*n* = 6, 31.6%). Three patients had an extended hospitalization period, but none died or had functional impairment due to sugammadex-induced anaphylaxis. We know that 18 patients fully recovered after sugammadex-induced anaphylaxis. The remaining patient’s records were missing.

### 3.2. Case Reports Published by Korean Researchers

Five relevant case reports were published between 2013 and 2020. One case report published in 2020 was submitted to a journal in 2019. Table 2 presents a summary of the reports on sugammadex-induced anaphylaxis [14,15,16,17,18]. None of the five patients had previously been exposed to sugammadex. A skin prick test or intradermal skin test were performed in two and three cases, respectively. In all cases, the time from the onset of anaphylaxis to hemodynamic recovery was less than 2 h, and the patients did not experience any complications, and none died. All patients in the aforementioned five case reports were matched with patients from the KIDS-KAERS database by age, sex, and year in which sugammadex-induced anaphylaxis occurred.

### 3.3. Single-Center Retrospective Cohort Study

**Figure 1:**
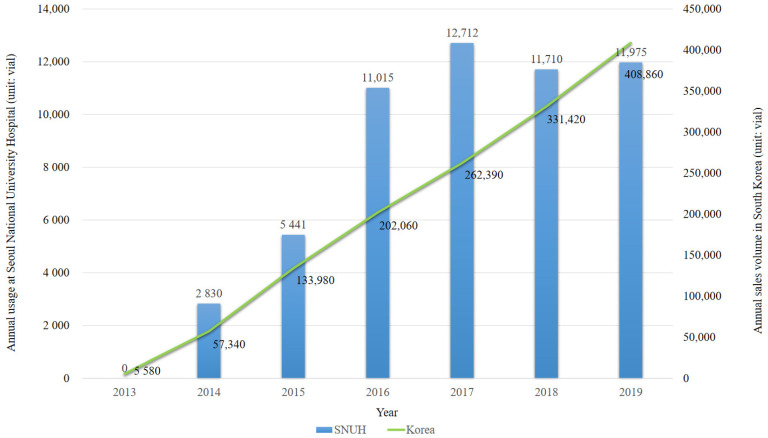
Annual usage of sugammadex in Seoul National University hospital (SNUH) and annual sales volume of sugammadex in South Korea (unit: vial).

Sugammadex has been prescribed in our institution since 2014, and its prescription volume showed an increasing trend over the study period (Figure 1). During the study period, 226,452 surgeries were performed under general anesthesia at our institution, and 69,362 vials of sugammadex were administered to 52,812 adult patients in 60,140 surgeries. None of the patients were labeled as having a drug allergy to sugammadex in the EMR-based surveillance system. In 1697 surgeries, sugammadex and epinephrine were intravenously administered within the same anesthetic record, and no case of sugammadex-induced anaphylaxis was identified. During the same period, 11 patients (4.9/100,000; 95% confidence interval (CI): 2.7–8.8/100,000) were identified with rocuronium-induced anaphylaxis through the EMR-based surveillance system.

## 4. Discussion

In this study, we investigated the incidence of sugammadex-induced anaphylaxis in South Korea. The main findings of the present study are as follows: in the national pharmacovigilance database, 19 cases of sugammadex-induced anaphylaxis were reported, but no patients died or experienced functional impairment due to it. We also investigated its incidence in our institution: there was no case of sugammadex-induced anaphylaxis in the 60,140 cases where sugammadex was administered. Our results provide additional evidence for the safety of sugammadex in the Korean population.

Several large retrospective studies have been conducted in Japan and the UK. In a single-center retrospective study conducted in Japan, the incidence of sugammadex-induced anaphylaxis was 0.039% (6 of 15,479 cases) [11]. Another multicenter retrospective study in Japan reported an incidence of 0.02% (6 of 29,962 exposures) [6]. However, a nationwide survey on perioperative anaphylaxis in the UK found only one case out of 64,121 exposures (0.0016%) [10]. Recently, a single-center retrospective study in the US reported an incidence of 0.009% (2 cases out of 23,446 exposures) [12]. The large differences in the incidence rates among studies can be attributed to the differences in the diagnostic criteria or reporting systems.

In this study, we did not find cases of sugammadex-induced anaphylaxis in our institution cohort; however, this cannot be attributed to underreporting in our EMR-based surveillance system as we identified 11 cases of rocuronium-induced anaphylaxis using the same search protocol. The incidence of rocuronium-induced anaphylaxis in this data was calculated at 4.9/100,000, which is in the range of that reported in previous studies [24,25].

Although it was difficult to estimate the exact overall incidence of sugammadex-induced anaphylaxis due to the inaccuracy of both the denominator (number of sugammadex exposure cases during the study period) and numerator (number of sugammadex-induced anaphylaxis cases during the study period), we estimated its incidence using several assumptions. Assuming that the reporting or efficiency was 10% 50%, the estimated incidence of sugammadex-induced anaphylaxis was 0.0135% or 0.0027%, and they were in the range of the incidence reported in the multicenter retrospective study in Japan (0.02%) [6] and the nationwide survey in the UK (0.0016%) [10], respectively. The result of the latter calculation can explain why no cases of sugammadex-induced anaphylaxis were found in the 60,140 surgical cases in our institution.

As per our national pharmacovigilance database, none of the 19 patients with sugammadex-induced anaphylaxis died or experienced functional disability after the event, which is consistent with the results of previous studies on sugammadex-induced anaphylaxis [6,11,12]. However, this might be due to the lack of a sufficient number of subjects, considering the low mortality rate of perioperative anaphylaxis. In a national survey of UK anesthesiologists, the mortality rate of severe perioperative anaphylaxis was reported to be 2.3% [26]. In the 264 cases of perioperative anaphylaxis from the West Australian Anesthetic Mortality Committee database, there were no deaths, and the estimated CI of its mortality rate was reported to be within the range of 0–1.4% [27]. In the 273 cases of sugammadex-induced anaphylaxis identified in the post-marketing database, four deaths were reported (mortality rate, 1.47%; 95% CI: 0.40–3.72%) [9]. Therefore, anesthesiologists should always be cautious of the fatal risk of anaphylaxis when administering sugammadex.

Another notable finding of our study was that rocuronium was the most frequent other culprit drug. Although we did not investigate the causal relationship due to the limited information, rocuronium can act as the cause of sugammadex-induced anaphylaxis. The first case report of anaphylaxis caused by the sugammadex-rocuronium complex was reported in 2016 [28]. Several cases of anaphylaxis caused by the sugammadex-rocuronium complex have since been reported (one of them is included in Table 1) [14,29,30]. Structural changes caused by the combination of sugammadex and rocuronium can act independently as allergens that can cause anaphylaxis [30]. In all of these cases, skin tests with each drug revealed negative results, but skin tests with the pre-mixed sugammadex-rocuronium complex showed positive results. Therefore, when anaphylaxis by sugammadex is suspected, allergy tests should be conducted with the sugammadex-rocuronium complex as well as sugammadex alone.

Although anaphylaxis is an unpreventable ADR, information regarding the incidence of this serious ADR is important to ensure patient safety. In South Korea, the manufacturer’s patent for sugammadex expires on 12 April 2022, and its use in general anesthesia is expected to increase significantly thereafter. Accordingly, the number of cases of sugammadex-induced anaphylaxis will also gradually increase. However, it is difficult to investigate its incidence prospectively because of the very low incidence. Therefore, a high-quality retrospective study with reliable data is required, which will require the effort and attention of anesthesiologists who should be the main actors in ADR reporting.

The results of our study should be interpreted with caution. First, a major limitation of this study is the inherent limitation of the retrospective study design. Our data sources lacked important clinical details, such as patient-related factors, severity of anaphylaxis reactions, skin test results, and treatments for anaphylaxis. We had to rely on the reporter’s diagnosis of anaphylaxis. Second, due to the aforementioned limitations, the estimated prevalence was based on several assumptions. Third, the mechanism of sugammadex-induced anaphylaxis could not be explored. Further studies are needed to outline the underlying mechanisms. Despite these limitations, to the best of our knowledge, this is the first study of sugammadex-induced anaphylaxis in South Korea.

In conclusion, we identified 19 cases of sugammadex-induced anaphylaxis from the national pharmacovigilance database of South Korea and none from our EMR database of 60,140 cases where sugammadex was administered. Although the incidence of sugammadex-induced anaphylaxis calculated based on our nationwide database is likely to be underestimated, owing to the limitations of self-reported data, its incidence is not a pressing issue at present compared to the risks posed by other drugs, and its use in general anesthesia should not be hindered.

## Figures and Tables

**Table 1:** Summary of baseline characteristics and anaphylaxis-related information in patients with anaphylaxis to sugammadex in the Korea Institute of Drug Safety & Risk Management-Korea Adverse Event Reporting System database.

	Total *n* = 19
Age	58 (38–62)
Sex (Male/Female)	14 (73.7)/5 (26.3)
Year of event (2014/2015/2016/2017/2018/2019)	1/0/6/1/1/10
History of drug allergy/Asthma	2 (10.5)/1 (5.3)
Diagnosis	
Anaphylactic shock	10 (52.6)
Anaphylaxis	7 (36.8)
Anaphylactic reaction	2 (10.5)
Dosage of sugammadex	
100 mg	3 (15.8)
200 mg	9 (47.4)
Not recorded	7 (36.8)
Other suspected drugs	
Rocuronium	6 (31.6)
Ceftriaxone	1 (5.3)
Fentanyl	1 (5.3)
Glycopyrronium bromide	1 (5.3)
Iohexol	1 (5.3)
Pyridostigmine	1 (5.3)
Level of seriousness	
Prolonged admission	3 (15.8)
Life-threatening complication	3 (15.8)
Disability for functional decline of severe degree	0
Death	0
Recovery status	
Recovered/Not recorded	18 (94.7)/1 (5.3)
Subject of reporting	
Doctor	8 (42.1)
Pharmacist	4 (21.1)
Nurse	5 (26.3)
Others	2 (10.5)

Values are expressed as number (%) or median (interquartile range).

**Table 2:** Summary of sugammadex-induced anaphylaxis case reports published by Korean authors.

Study	Age (year)	Sex	Height (cm)	Weight (kg)	History of Allergy	Sugammadex Dose	Onset of Reaction (min)	Time to Achieve Hemodynamic Stability (min)	Skin Prick Test	Intradermal Skin Test
Kim et al. [14]	42	Male	175	78	Cat hair	2.5 mg/kg	NA	80	Not performed	Positive for sugammadex-rocuronium complex
Koo et al. [15]	60	Male	160	61.4	No	200 mg	2	20	Positive	Positive (1:100–10,000)
Choi et al. [16]	60	Male	159	64	No	200 mg	3 min after extubation	30	Not performed	Positive (1:100–1000)
Hwang et al. [17]	69	Female	158	50	No	100 mg	8	70	Not performed	Not performed
Yoo et al. [18]	35	Male	182	109	Animal hair	200 mg	NA	10	Weakly positive	Not performed

ASA, American Society of Anesthesiologist.

## Data Availability

Not applicable.

## References

[1] Keating G.M. (2016). Sugammadex: A review of neuromuscular blockade reversal. Drugs.

[2] Abad-Gurumeta A., Ripollés-Melchor J., Casans-Francés R., Espinosa A., Martínez-Hurtado E., Fernández-Pérez C., Ramírez J.M., Lõpez-Timoneda F., Calvo-Vecino J.M. (2015). A systematic review of sugammadex vs neostigmine for reversal of neuromuscular blockade. Anaesthesia.

[3] Kheterpal S., Vaughn M.T., Dubovoy T.Z., Shah N.J., Bash L.D., Colquhoun D.A., Shanks A.M., Mathis M.R., Soto R.G., Bardia A. (2020). Sugammadex versus neostigmine for reversal of neuromuscular blockade and postoperative pulmonary complications (STRONGER): A multicenter matched cohort analysis. Anesthesiology.

[4] Deljou A., Schroeder D.R., Ballinger B.A., Sprung J., Weingarten T.N. (2019). Effects of sugammadex on time of first postoperative bowel movement. Mayo Clin. Proc. Innov. Qual. Outcomes.

[5] Oh T.K., Oh A.Y., Ryu J.H., Koo B.W., Song I.A., Nam S.W., Jee H.J. (2019). Retrospective analysis of 30-day unplanned readmission after major abdominal surgery with reversal by sugammadex or neostigmine. Br. J. Anaesth..

[6] Orihara M., Takazawa T., Horiuchi T., Sakamoto S., Nagumo K., Tomita Y., Tomioka A., Yoshida N., Yokohama A., Saito S. (2020). Comparison of incidence of anaphylaxis between sugammadex and neostigmine: A retrospective multicentre observational study. Br. J. Anaesth..

[7] The Development and Regulatory History of Sugammadex in the United State—Anesthesia Patient Safety Foundation. https://www.apsf.org/article/the-development-and-regulatory-history-of-sugammadex-in-the-united-states/.

[8] Min K.C., Bondiskey P., Schulz V., Woo T., Assaid C., Yu W., Reynders T., Declercq R., McCrea J., Dennie J. (2018). Hypersensitivity incidence after sugammadex administration in healthy subjects: A randomised controlled trial. Br. J. Anaesth..

[9] Min K.C., Woo T., Assaid C., McCrea J., Gurner D.M., Sisk C.M.C., Adkinson F., Herring W.J. (2018). Incidence of hypersensitivity and anaphylaxis with sugammadex. J. Clin. Anesth..

[10] Harper N.J.N., Cook T.M., Garcez T., Farmer L., Floss K., Marinho S., Torevell H., Warner A., Ferguson K., Hitchman J. (2018). Anaesthesia, surgery, and life-threatening allergic reactions: Epidemiology and clinical features of perioperative anaphylaxis in the 6th National Audit Project (NAP6). Br. J. Anaesth..

[11] Miyazaki Y., Sunaga H., Kida K., Hobo S., Inoue N., Muto M., Uezono S. (2018). Incidence of anaphylaxis associated with sugammadex. Anesth. Analg..

[12] Burbridge M.A. (2020). Incidence of Anaphylaxis to Sugammadex in a Single-Center Cohort of 19,821 Patients. Anesth. Analg..

[13] MSD Korea Inc. Pharmacopoeia of Bridion^®^. https://www.msd-korea.com/product-and-disease/product-info/home.html#nojs.

[14] Kim G.H., Choi W.S., Kim J.E., Yun M.J., Koo M.S., Kwon M., Seo H. (2019). Anaphylactic shock after sugammadex administration, induced by formation of a sugammadex-rocuronium complex: A case report. Korean J. Anesthesiol..

[15] Koo B.S., Lee S.J., Na H.W., Kang W.B., Kim S.H. (2019). A suspected sugammadex-induced anaphylactic shock—A case report. Anesth. Pain Med..

[16] Choi S.C., Han S., Kwak J., Lee J.Y. (2020). Anaphylaxis induced by sugammadex and sugammadex-rocuronium complex—A case report. Korean J. Anesthesiol..

[17] Hwang M., Won Y.J., Lee I.-O., Koo E.H., Jung W. (2015). A suspected case of sugammadex-induced anaphylactic shock—A case report. Anesth. Pain Med..

[18] Yoo J.H., Kim S.I., Ok S.Y., Park S.Y., Cho A., Han Y.M., Jun M.R. (2016). Suspected anaphylactic reaction associated with sugammadex—A case report. Korean J. Anesthesiol..

[19] Park J.W., Park K.H., Lee S.C., Yuk J.E., Kim S.R., Lee J.H. (2019). Eperisone-induced anaphylaxis: Pharmacovigilance data and results of allergy testing. Allergy Asthma Immunol. Res..

[20] FDA Advisory Committee (2015). NDA 22225: Sugammadex Injection Anesthetic and Analgesic Drug Products Advisory Committee (AC) Meeting 6 November 2015 Sugammadex AC Briefing Document. https://www.fdanews.com/ext/resources/files/11-15/110615-merck.pdf?1520874794.

[21] Simons F.E.R., Ardusso L.R.F., Bilò M.B., El-Gamal Y.M., Ledford D.K., Ring J., Sanchez-Borges M., Senna G.E., Sheikh A., Thong B. (2011). World allergy organization guidelines for the assessment and management of anaphylaxis. World Allergy Organ. J..

[22] World Health Organization (WHO)—Uppsala Monitoring Centre The Use of the WHO-UMC System for Standardized Case Causality Assessment. https://www.who.int/medicines/areas/quality_safety/safety_efficacy/WHOcausality_assessment.pdf.

[23] Heeley E., Riley J., Layton D., Wilton L.V., Shakir S.A.W. (2001). Prescription-event monitoring and reporting of adverse drug reactions. Lancet.

[24] Cho Y.J., Ju J.W., Sim H., Lee J.H., Hong D.M., Kim T.K., Min J.J., Song W.J., Kang H.R., Cho S.H. (2016). Intraoperative anaphylaxis to neuromuscular blocking agents: The incidence over 9 years at two tertiary hospitals in South Korea. Eur. J. Anaesthesiol..

[25] Sadleir P.H.M., Clarke R.C., Bunning D.L., Platt P.R. (2013). Anaphylaxis to neuromuscular blocking drugs: Incidence and cross-reactivity in Western Australia from 2002 to 2011. Br. J. Anaesth..

[26] Kemp H.I., Cook T.M., Thomas M., Harper N.J.N. (2017). UK anaesthetists’ perspectives and experiences of severe perioperative anaphylaxis: NAP6 baseline survey. Br. J. Anaesth..

[27] Gibbs N.M., Sadleir P.H., Clarke R.C., Platt P.R. (2013). Survival from perioperative anaphylaxis in western australia 2000–2009. Br. J. Anaesth..

[28] Ho G., Clarke R.C., Sadlelr P.H.M., Platt P.R. (2016). The first case report of anaphylaxis caused by the inclusion complex of rocuronium and sugammadex. A A Case Rep..

[29] Yamaoka M., Deguchi M., Ninomiya K., Kurasako T., Matsumoto M. (2017). A suspected case of rocuronium–sugammadex complex-induced anaphylactic shock after cesarean section. J. Anesth..

[30] Ebo D.G., Baldo B.A., Van Gasse A.L., Mertens C., Elst J., Sermeus L., Bridts C.H., Hagendorens M.M., De Clerck L.S., Sabato V. (2020). Anaphylaxis to sugammadex-rocuronium inclusion complex: An IgE-mediated reaction due to allergenic changes at the sugammadex primary rim. J. Allergy Clin. Immunol. Pract..

